# Supplementary material for: Effectiveness and Safety of Ayurvedic Medicines in Type 2 Diabetes Mellitus Management: A Systematic Review and Meta-Analysis
Source: Front Pharmacol. 2022 Jun 8;13:821810. doi: 10.3389/fphar.2022.821810 (PMC9213670; doi:10.3389/fphar.2022.821810)
Supplement: Supplementary file 1 [file DataSheet1.zip › Supplementary Material/Forest and Funnel Plots/Gynostemma pentaphyllum/Gynostemma pentaphyllum.pdf]

Outcomes	Number of studies	Mean Diff. with 95% CI	Heterogeneity (%)
HbA1c	2	-1.01 [-1.45, -0.56]	0.00
FBG	2	-29.03 [-43.45, -14.61]	23.10
PPBG	2	-79.93 [-134.40, -25.46]	0.00
Fasting insulin*	2	-3.51 [-8.00, 0.99]	0.00
Stimulated insulin*	2	-2.99 [-25.59, 19.61]	57.71
Fasting C-peptide*	1	0.00 [-0.81, 0.81]	N/A
Stimulated C-peptide*	1	-0.50 [-1.84, 0.84]	N/A
Insulin resistance	2	-2.21 [-4.42, 0.00]	0.00
Body weight	2	1.68 [-3.58, 6.93]	0.00
BMI	2	0.58 [-0.90, 2.06]	0.00
Waist circumference	1	-1.50 [-8.28, 5.28]	N/A
SBP	1	-2.50 [-9.92, 4.92]	N/A
DBP	1	-2.30 [-7.48, 2.88]	N/A
TC	1	23.20 [-5.60, 52.00]	N/A
HDL-C	1	-3.87 [-11.67, 3.93]	N/A
LDL-C	1	23.20 [5.00, 41.40]	N/A
TG	1	26.55 [-61.32, 114.42]	N/A

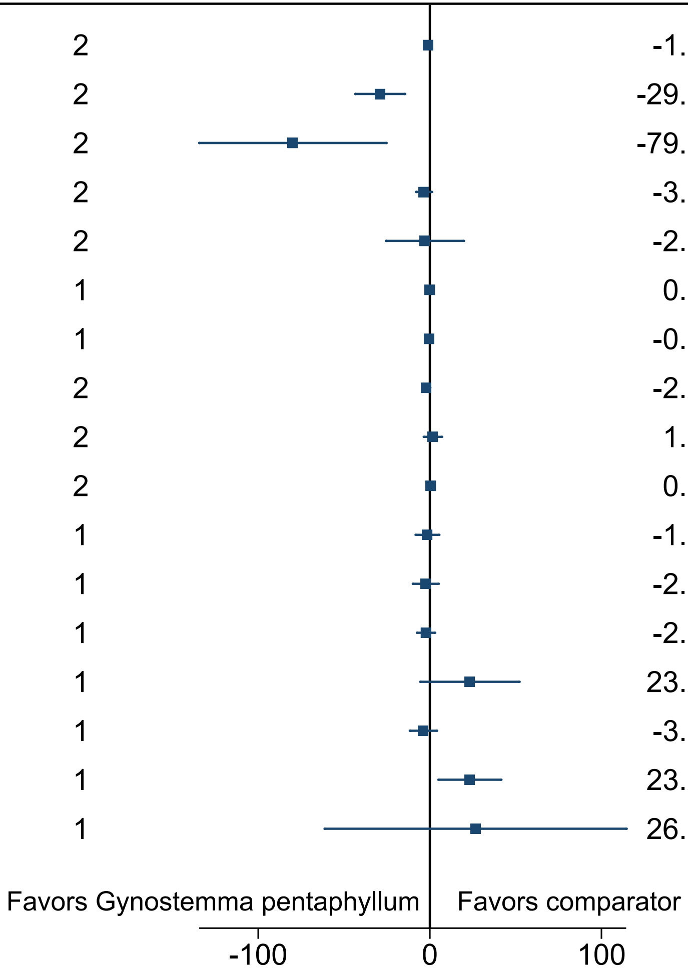

*Favors intervention/comparator as insulin sensitizer
